# Orchestration of Ion Channels and Transporters in Neocortical Development and Neurological Disorders

**DOI:** 10.3389/fnins.2022.827284

**Published:** 2022-02-14

**Authors:** Yuki Bando, Masaru Ishibashi, Satoru Yamagishi, Atsuo Fukuda, Kohji Sato

**Affiliations:** ^1^Department of Organ and Tissue Anatomy, Hamamatsu University School of Medicine, Hamamatsu, Japan; ^2^Department of Neurophysiology, Hamamatsu University School of Medicine, Hamamatsu, Japan

**Keywords:** cerebral cortex, neurogenesis, neuronal migration, dendrite, axon, ion channel, transporter, channelopathy

## Abstract

Electrical activity plays crucial roles in neural circuit formation and remodeling. During neocortical development, neurons are generated in the ventricular zone, migrate to their correct position, elongate dendrites and axons, and form synapses. In this review, we summarize the functions of ion channels and transporters in neocortical development. Next, we discuss links between neurological disorders caused by dysfunction of ion channels (channelopathies) and neocortical development. Finally, we introduce emerging optical techniques with potential applications in physiological studies of neocortical development and the pathophysiology of channelopathies.

## Introduction

Precise formation of neocortical circuits is essential for brain function. The cerebral cortex consists of six layers. Its laminar structure is formed in an “inside-out” manner; layer 6 is formed first, followed by formation of upper layers above the lower layers. Neocortical excitatory neurons are produced from neural progenitor cells in the ventricular zone (VZ). During neurogenesis, intermediate progenitors are produced from radial glia. Intermediate progenitors then produce or differentiate into excitatory neurons (Hevner, 2006). The newly born neurons migrate toward the marginal zone (MZ).

During migration, neurons dynamically change their morphology. Neocortical excitatory neurons slowly move in the subventricular zone (SVZ) and the intermediate zone (IZ) with small processes in multiple directions (multipolar migration) (Tabata and Nakajima, 2003). Then, migrating neurons change their shape at the border between the IZ and the cortical plate (CP) to a bipolar shape with long leading processes and short trailing processes, and migrate along the radial axis toward the cortical surface (Nadarajah et al., 2003). Finally, neurons stop migration below the MZ, and elongate dendrites and axons (Tissir and Goffinet, 2003; Mizuno et al., 2007, 2014). The molecular mechanisms of neocortical development have been intensely studied (Tessier-Lavigne and Goodman, 1996; O’Leary and Nakagawa, 2002; Hevner, 2006; Molyneaux et al., 2007; Kawauchi and Hoshino, 2008; Kawauchi, 2012; Marín, 2012). As well as genetic programs, electrical activity and Ca^2+^ signaling are also crucial for these processes (Katz and Shatz, 1996; Spitzer, 2006). Recent reports showed that dysfunction of ion channels or transporters disrupts neocortical development by altering electrical properties and Ca^2+^ signaling and may be linked to neurological disorders (Kullmann, 2010; Schmunk and Gargus, 2013; Guglielmi et al., 2015; Heyes et al., 2015; Kahle et al., 2016). In this review, we summarize how ion channels and transporters regulate electrical properties and Ca^2+^ signaling during neocortical development, focusing on excitatory neurons. Next, we discuss possible links between abnormal electrical signaling caused by dysfunction of ion channels or transporters and neurological disorders. Finally, we discuss the potential application of emerging optical techniques to address remaining issues related to the physiological mechanisms of neocortical development and the pathophysiology of channelopathies *in vivo*.

### Electrical Signaling During Neocortical Development

**FIGURE 1:**
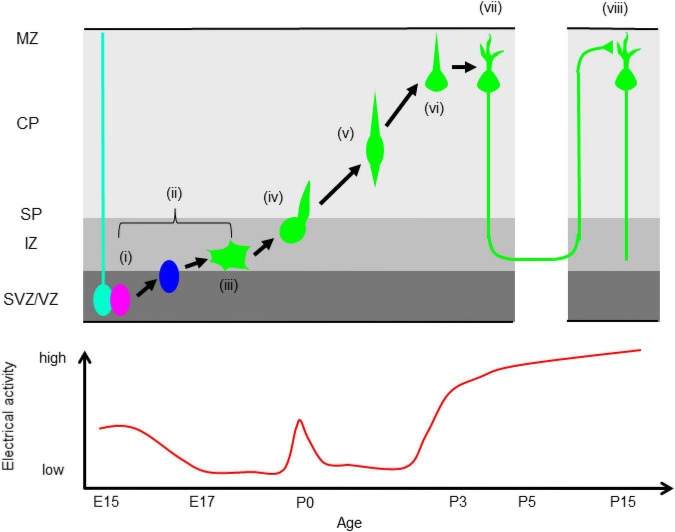
Correlation between electrical activity and neocortical development. (i) Proliferation, (ii) neuronal differentiation, (iii) multipolar migration (iv) multipolar-to-bipolar transition, (v) radial migration, (vi) termination of migration, (vii) dendrite and axonal elongation, (viii) synapse formation. VZ, ventricular zone, SVZ, subventricular zone, IZ, intermediate zone, SP, subplate, CP, cortical plate, MZ, marginal zone. The bottom panel shows temporal changes of electrical activity. Electrical activity is high during neurogenesis, low during migration except in the boundary between the IZ and SP, and is elevated again after neurons reach the MZ.

The roles of electrical signaling in axonal and dendritic growth and remodeling during late developmental stages have been intensely studied (Katz and Shatz, 1996; Price et al., 2006). Further studies revealed that electrical signaling is also crucial for early cortical development including neuronal proliferation, differentiation, and migration (Spitzer, 2006). These studies suggest that temporal regulation of electrical signals is critical for neocortical development (Figure 1). We discuss the details below.

#### Neurogenesis, Differentiation, and Cell Fate Specification

Radial glial cells express various ion channels such as α-amino-3-hydroxy-5-methyl-4-isoxazolepropionic acid (AMPA) and kainate type glutamate receptor, γ-aminobutyric acid type A receptor (GABA_*A*_R), voltage-gated Ca^2+^ channels (VGCCs), P2X receptor, and connexin 26 and 43, but not N-methyl-D-aspartate (NMDA) type glutamate receptor (LoTurco et al., 1995; Bittman and LoTurco, 1999). The electrical properties of neural progenitors are distinct from those of mature neurons (Liu et al., 2010). Neural progenitors are non-spiking because of a small voltage-dependent Na^+^ current. Their resting potential is about –75 mV, and their input resistance is about 350 MΩ. Activation of AMPA and kainate receptor and GABA_*A*_R inhibits DNA synthesis by depolarizing membrane potentials (LoTurco et al., 1995). Further studies revealed that Ca^2+^ transients are required for the transition from G1 to S phase by releasing adenosine triphosphate (ATP) from progenitor cells through gap junction/hemichannels resulting in activation of P2X receptors. This indicates that temporal patterns of Ca^2+^ signaling are critical for cell cycle progression and neurogenesis (Weissman et al., 2004; Liu et al., 2010). A recent study demonstrated that activation of GABA_*A*_R promotes the transition from apical to basal progenitor cells by elevating of the intracellular Ca^2+^ concentration, suggesting that the excitatory action of GABAergic signals regulates differentiation of neural progenitors (Tochitani et al., 2021).

Interestingly, electrical activity also affects the cell fate of cortical excitatory neurons. A gain-of-function mutation of an L-type VGCC, CACNA1C (Ca_*v*_1.2) reduces the fraction of Satb2-positive callosal projection neurons and increases the fraction of Ctip2-positive corticofugal projection neurons in layer 5 (Paşca et al., 2011). Recently, Vitali et al. (2018) showed that regulation of the resting potential is important for specification of upper layer neurons. Neural progenitors are more hyperpolarized at embryonic day 15 (E15) than at E14, and premature hyperpolarization of progenitors by expression of an inward rectifier K^+^ channel, KCNJ2 (Kir2.1), decreases the fraction of RORβ-positive layer 4 neurons and increases the fraction of Brn2-positive layer 2/3 neurons. There remain interesting questions about how electrical signals regulate transcription networks and how plastic production of neuronal populations is during cortical development.

#### Neuronal Migration

Newly born neurons have a more depolarized resting potential than neural progenitors (∼ –60 mV), drastically increased input resistance (∼ 3 GΩ), and less frequent spontaneous Ca^2+^ transients in the SVZ and IZ (Figure 1; Bando et al., 2014). Immature neurons express GABA_*A*_R, NMDAR, CACNA1C, and CACNA1D (Ca_*v*_1.3). The expression levels of CACNA1C and CACNA1D are higher in the IZ and CP than in the VZ (Kamijo et al., 2018; Horigane et al., 2021). In the IZ, glutamate promotes migration into the CP via NMDAR (Behar et al., 1999). Around the border between the IZ and subplate (SP), migrating excitatory neurons show more frequent and larger Ca^2+^ transients than neurons in the lower IZ (Figure 1), because of activation of NMDAR by SP neurons. The increase of Ca^2+^ transients promotes the multipolar-to-bipolar transition of migrating upper layer neurons at E17 and E18 (Ohtaka-Maruyama et al., 2018; Horigane et al., 2021). During locomotion in the CP, the frequency of spontaneous Ca^2+^ transients decreases, and migrating neurons show more frequent Ca^2+^ transients after reaching the MZ (Figure 1; Bando et al., 2014, 2016). Suppression of spontaneous activity by blocking GABA_*A*_R results in acceleration of radial migration and invasion of neurons into the MZ (Behar et al., 2000; Heck et al., 2007; Furukawa et al., 2014). A tandem pore domain K^+^ channel, KCNK9 (K_2P_9.1) promotes migration by suppressing spontaneous Ca^2+^ transients (Bando et al., 2014). Nakagawa-Tamagawa et al. (2021) reported that a disease-associated mutation of CACNA1C causes migration arrest. Furthermore, the strong elevation of spontaneous activity during early developmental stages in the neocortex stops neuronal migration, and induces dendritic branch formation (Bando et al., 2016). These results show that spontaneous Ca^2+^ transients should be kept low during radial migration and that an increase of Ca^2+^ signaling acts as a stop signal in cortical excitatory neurons. Electrical signals induce elevation of intracellular Ca^2+^, which functions as a second messenger; it activates multiple Ca^2+^-dependent enzymes, followed by activation of downstream signaling cascades, and also regulates cytoskeletal dynamics and exocytosis. Taken together, these findings show that properly regulated Ca^2+^ signaling at each developmental stage is critical for neocortical formation (Manent and Represa, 2007; Zheng and Poo, 2007; Uhlén et al., 2015; Horigane et al., 2019; Medvedeva and Pierani, 2020).

The correlation between the intracellular Ca^2+^ level and migration speed differs among cell types. Komuro and Rakic (1996) and Kumada and Komuro (2004) showed that loss of spontaneous Ca^2+^ transients is a stop signal for cerebellar granule cell migration. Similar to cerebellar granule cells, neocortical inhibitory interneurons stop migration in the absence of spontaneous Ca^2+^ transients caused by excitatory-to-inhibitory switching of GABAergic signaling (Bortone and Polleux, 2009). Interestingly, migration of neocortical excitatory neurons is also regulated in a Ca^2+^-dependent manner, but with the opposite mechanism as described above. It remains unclear what underlies the difference in Ca^2+^-dependency between migration of cortical excitatory and cortical inhibitory interneurons/cerebellar granule cells.

#### Dendrite Formation, Axonal Projection, and Synapse Formation

Post-migratory neurons become electrically mature; expression of voltage-gated Na^+^ channels increases (peak Na^+^ current: ∼ –90 pA at P0, and ∼ –800 pA at P4), and neurons start firing action potentials (Picken Bahrey and Moody, 2003). Their input resistance is significantly reduced (0.6–1.6 GΩ at P4). Activity-dependent formation and remodeling of dendrites, axons, and synapses have been intensely studied in multiple systems such as visual, somatosensory, olfactory, and motor systems (Hubel et al., 1977; Iwasato et al., 1997; Wong and Ghosh, 2002; Hanson and Landmesser, 2004; Serizawa et al., 2006). Electrical activity is crucial for projection and arborization of thalamocortical axons (Antonini and Stryker, 1993; Uesaka et al., 2007; Mire et al., 2012; Antón-Bolaños et al., 2019). Mire et al. (2012) reported that temporal patterns of thalamocortical neuron activity are crucial for axon guidance through regulation of the axon guidance molecule, Robo1. The activity of thalamocortical axons affects spatial patterning of dendrites in layer 4 neurons through activation of NMDAR (Mizuno et al., 2014). This demonstrates that the cooperative activity of pre- and postsynaptic neurons shapes the thalamocortical circuit (Yamada et al., 2010; Mizuno et al., 2014). Excitatory GABA is essential for dendrite formation in layer 2/3 pyramidal neurons. In layer 2/3 pyramidal neurons, excitatory-to-inhibitory switching of GABA occurs between postnatal day 6 (P6) and P14. Premature excitatory-to-inhibitory switching of GABA by expressing K-Cl co-transporter 2 (KCC2) suppresses dendritic growth in layer 2/3 pyramidal neurons (Cancedda et al., 2007). Suppression of neural activity by expressing KCNJ2 significantly reduces dendritic growth, and layer-specific projection of callosal axons in cortical layer 2/3 neurons (Cancedda et al., 2007; Mizuno et al., 2007, 2010; Wang et al., 2007). Expression of a gain-of-function CACNA1C mutant also disrupts callosal axon projection (Nakagawa-Tamagawa et al., 2021). These reports suggest that the optimal frequency of electrical activity is critical for callosal axon projection. A further study revealed that layer-specific projection of callosal axons requires postsynaptic activity (Mizuno et al., 2010).

### Potential Links Between Dysfunction of Ion Channels/Transporters and Neurological Disorders

Dysfunction of ion channels or transporters is associated with neurological and psychiatric disorders such as epilepsy, autism spectrum disorder, and schizophrenia (Kullmann, 2010; Schmunk and Gargus, 2013; Guglielmi et al., 2015; Heyes et al., 2015). In some patients and mouse models of channelopathies, malformations of cortical development are observed. Ion channels and transporters play crucial roles in neocortical development; therefore, developmental defects might underlie the symptoms of channelopathies. We describe some examples below.

NMDAR is a key ligand-gated ion channel for any developmental events and plasticity in the nervous system. Mutations of NMDAR are associated with a wide variety of neurological and psychiatric disorders such as schizophrenia, epilepsy, and depression (Kalia et al., 2008; Hardingham and Do, 2016; Adell, 2020).

Tandem pore domain K^+^ channels suppress neuronal excitability by hyperpolarizing the resting membrane potential and reducing membrane resistance. A dominant-negative mutation of KCNK9 was found in patients with Birk-Barel syndrome, a maternally transmitted genomic imprinting disorder characterized by severe intellectual disability, hypotonia, and dysmorphism in the form of an elongated face (Barel et al., 2008). Knock-down or functional blockade of KCNK9 by expressing a disease-associated dominant-negative mutant channel impairs neuronal migration in the developing neocortex (Bando et al., 2014). Since migration defect is associated with many neurological and psychiatric disorders (Ross and Walsh, 2001; LoTurco and Bai, 2006; Ben-Ari, 2008), migration defect might be a candidate of its pathogenesis. Interestingly, another tandem pore domain K^+^ channel, KCNK2 (K_2P_2.1) might be linked to brain aging. Le Guen et al. (2019) investigated the genetic influence on sulcal widening in elderly individuals. They found that the regulatory region of KCNK2 influences sulcal widening, suggesting a potential link between KCNK2 expression and brain atrophy (Le Guen et al., 2019).

CACNA1C, a L-type VGCC, is associated with Timothy syndrome, which is characterized by long QT syndrome in the heart, autism spectrum disorder, and mild dysmorphism of the face. Several gain-of-function mutations of CACNA1C have been found in patients (Heyes et al., 2015). Disease-associated mutant CACNA1C disrupts neocortical development, including cell fate specification of cortical projection neurons, radial migration, dendrite formation/remodeling, and callosal axon projection (Paşca et al., 2011; Kamijo et al., 2018; Horigane et al., 2021; Nakagawa-Tamagawa et al., 2021). Downregulation of CACNA1C is also associated with psychiatric disorders. A loss-of function mutation and lower expression level of CACNA1C were found in patients with schizophrenia by genome-wide screening of disease-associated mutations (Purcell et al., 2014; Roussos et al., 2014; Heyes et al., 2015). Conditional knockout of CACNA1C impairs neurite growth in cultured cortical neurons (Kamijo et al., 2018); therefore, downregulation of CACNA1C might cause psychotic symptoms by disrupting neocortical development.

Excitatory-to-inhibitory switching of GABA is mediated by a change in expression of Cl^–^ transporters. During the early developmental stage, Na-K-Cl co-transporter 1 (NKCC1), which transports Cl^–^ into the cell, is highly expressed. In the later stage, expression of NKCC1 decreases and expression of KCC2, which transports Cl^–^ out of the cell, is elevated. Excitatory-to-inhibitory switching of GABA plays important roles in neocortical development. Excitatory GABA regulates neuronal production, migration, and dendrite formation (Cancedda et al., 2007; Heck et al., 2007; Tochitani et al., 2021). Dysfunction of KCC2 or GABA_*A*_R is associated with epilepsy (Kaila et al., 2014; Kahle et al., 2016; Maljevic et al., 2019; Watanabe et al., 2019).

Similar to Timothy syndrome, mutations of ion channels associated with cardiac disorders can affect neocortical neural circuit formation. For example, expression of a gain-of-function KCNJ2 mutant that causes atrial fibrillation significantly reduces branching of callosal axons in the upper layers in the contralateral hemisphere (Mizuno et al., 2007).

In patients with other neurological channelopathies, malformation of cortical development was observed. Periventricular nodular heterotopia was observed in some patients with sleep-related hypermotor epilepsy and point mutations in the sodium-activated K^+^ channel KCNT1 (Slack or K_*Na*_1.1) (Rubboli et al., 2018). Polymicrogyria was observed in patients with drug-resistant epilepsy and mutations in the Ca^2+^-activated K^+^ channel KCNMA1 (BK channel) (Graber et al., 2021). Periventricular nodular heterotopia and focal cortical dysplasia were observed in patients with Dravet syndrome and mutations in the voltage-gated Na^+^ channel SCN1A (Na_*v*_1.1) (Barba et al., 2014). As discussed above, some neurological disorders are accompanied by malformation of the cortical gyrus. Genetically modified ferret and common marmoset are good experimental models to study the physiological mechanisms of gyrus formation (Sasaki et al., 2009; Kawasaki et al., 2012; Shinmyo et al., 2017). Despite intensive efforts in developmental and clinical studies, the links between developmental defects and channelopathies remain elusive. Further studies could reveal the developmental basis of neurological channelopathies.

## Discussion

### Future Perspectives: Potential Application of Advanced Optical Techniques in Developmental Neuroscience and Pathophysiological Studies of Neurological Disorders *in vivo*

To better understand the pathogenetic mechanisms of neurological channelopathies, it seems essential to investigate the roles of ion channels in neocortical development *in vivo*. Previously, developmental studies of the neocortex have been performed with fixed tissue and acute or cultured brain slices. Although these traditional methods are powerful tools to reveal the mechanisms of electrical activity-dependent neocortical development, there remain important problems. One of them is that secreted extracellular signals, including maternal signals, are washed out in the slice condition. For instance, taurine, a weak agonist of GABA_*A*_R, plays important roles in the development of the embryonic nervous system (Kilb and Fukuda, 2017). Taurine is provided to the embryo from the mother through the placenta because mouse embryos do not synthesize taurine (Sturman et al., 1977; Sturman, 1981). Thus, monitoring neocortical development in the intact brain is the next step. To achieve this, optical methods seem ideal. Recently, *in vivo* two-photon imaging of the neonatal and embryonic mouse neocortex has been achieved (Mizuno et al., 2014, 2018; Yuryev et al., 2016; Kawasoe et al., 2020; Hattori et al., 2020). Voltage imaging is promising to monitor electrical signals in the developing neocortex *in vivo* or *in utero*. Recently, the performance of genetically encoded voltage indicators (GEVIs) has been improved (Gong et al., 2015; Kannan et al., 2018; Adam et al., 2019; Bando et al., 2019a,b; Piatkevich et al., 2019; Villette et al., 2019; Cornejo et al., 2022). In contrast with chemical voltage-sensitive dyes (VSDs), GEVIs can be expressed in a cell-type-specific manner, resulting in an improved signal-to-noise ratio. Furthermore, long-term monitoring of electrical signals is possible using GEVIs, but not with patch-clamp recording and VSDs. Long-term monitoring of electrical activity would help researchers determine the correlation between electrical signals and developmental events such as neurogenesis, migration, and neurite growth. The combination of Ca^2+^ or voltage imaging and holographic photostimulation is a powerful tool to show causal links between electrical activity and developmental events (Carrillo-Reid et al., 2016). Two-photon multimodal imaging of voltage and Ca^2+^ in neuronal populations *in vivo* was recently reported (Bando et al., 2021). Application of these techniques could reveal how electrical signals are transformed into intracellular signals that drive neocortical circuit formation.

Developmental events occur in three-dimensional tissues. Thus, volumetric imaging is also important. Recently, fast three-dimensional imaging techniques were developed using a spatial light modulator, an acousto-optic lens, and an electrical tunable lens (Katona et al., 2012; Yang et al., 2016, 2018; Yang and Yuste, 2017). To image deep in the brain during development, three-photon imaging and an adaptive optics are also helpful (Ji et al., 2010; Horton et al., 2013). The combination of advanced microscopy and emerging optical probes could strongly drive developmental neuroscience.

Another important issue is how developmental defects cause neurological disorders. Recent studies showed that the properties of local neocortical circuits, such as neuronal ensembles (groups of co-active neurons), are altered in mouse models of psychiatric and neurological disorders, such as schizophrenia and autism (Fang and Yuste, 2017; Hamm et al., 2017). Simultaneous manipulation, and readout of cortical activity during behavior is promising to further elucidate the causal links between aberrant cortical activity and symptoms (Carrillo-Reid et al., 2016, 2019). Application of the recently developed two-photon mesoscope will help to clarify the alteration of cortex-wide computation at cellular resolution in animal models of disorders (Ota et al., 2021).

In summary, developmental studies revealed that dysfunction of ion channels and transporters disrupts neocortical circuit formation. Clinical studies reported potential links between neurological disorders and mutations of ion channels and transporters. However, the causal links between dysfunction of the ion channels and transporters, neocortical circuit formation, and neurological disorders are not understood. Emerging optical technologies could bridge these biophysical, developmental, and clinical studies.

## Author Contributions

YB organized the content. All authors wrote, revised, and approved the manuscript for publication.

## Conflict of Interest

The authors declare that the research was conducted in the absence of any commercial or financial relationships that could be construed as a potential conflict of interest.

## Publisher’s Note

All claims expressed in this article are solely those of the authors and do not necessarily represent those of their affiliated organizations, or those of the publisher, the editors and the reviewers. Any product that may be evaluated in this article, or claim that may be made by its manufacturer, is not guaranteed or endorsed by the publisher.
